# An evolutionary game model of individual choices and bed net use: elucidating key aspect in malaria elimination strategies

**DOI:** 10.1098/rsos.220685

**Published:** 2022-11-16

**Authors:** Laxmi, Calistus N. Ngonghala, Samit Bhattacharyya

**Affiliations:** ^1^ Disease Modelling Lab, Department of Mathematics, School of Natural Sciences, Shiv Nadar University, Gautam Buddha Nagar, India; ^2^ Department of Mathematics and Emerging Pathogens Institute, University of Florida, Gainesville, FL 32611, USA

**Keywords:** malaria, insecticide-treated nets, evolutionary game theory, social optimum

## Abstract

Insecticide-treated net (ITN) is the most applicable and cost-effective malaria intervention measure in sub-Saharan Africa and elsewhere. Although ITNs have been widely distributed to malaria-endemic regions in the past, their success has been threatened by misuses (in fishing, agriculture etc.) and decay in ITN efficacy. Decision-making in using the ITNs depends on multiple coevolving factors: malaria prevalence, mosquito density, ITN availability and its efficacy, and other socio-economic determinants. While ITN misuse increases as the efficacy of ITNs declines, high efficacy also impedes proper use due to free-riding. This irrational usage leads to increased malaria prevalence, thereby worsening malaria control efforts. It also remains unclear if the optimum ITN use for malaria elimination can be achieved under such an adaptive social learning process. Here, we incorporate evolutionary game theory into a disease transmission model to demonstrate these behavioural interactions and their impact on malaria prevalence. We show that social optimum usage is a function of transmission potential, ITN efficacy and mosquito demography. Under specific parameter regimes, our model exhibits patterns of ITN usage similar to observed data from parts of Africa. Our study suggests that the provision of financial incentives as prompt feedback to improper ITN use can reduce misuse and contribute positively towards malaria elimination efforts in Africa and elsewhere.

## 1. Introduction

Malaria is a mosquito-borne disease that affects 219 million people globally. It caused 435 000 deaths in 2017 and approximately 200 million (90%) of the reported cases in 2017 were from the World Health Organization (WHO) African region [1]. During the last two decades, several interventions have been used to prevent malaria transmission. These include vector interventions (e.g. insecticide-treated nets (ITNs), indoor residual spraying (IRS), larval control etc.), and intermittent preventive treatment for pregnant women [2,3]. Among these measures, ITNs have proven to be less costly, easy to use, and highly effective in preventing infection and reducing morbidity and mortality. ITNs protect humans from mosquito bites and the insecticide (pyrethroid) repels mosquitoes, thereby reducing their food-searching capabilities by disorienting them, or kills them. To avoid challenges including replacing ITNs every six months, a shift towards long-lasting insecticidal nets (LLINs) was endorsed by WHO [4]. Long-lasting insecticidal nets can keep their effectiveness for up to 3 years even after being washed many times.

**Figure 1.**
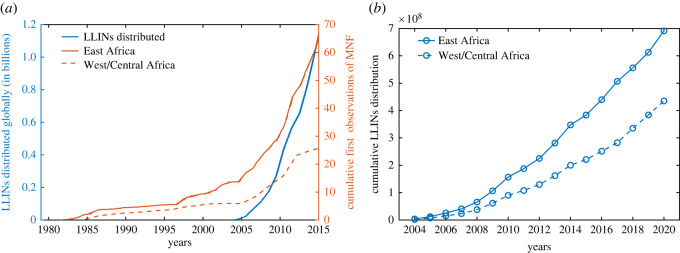
LLIN distributions and survey result of mosquito net fishing (MNF). (*a*) Cumulative first observations of MNF in East and West/Central Africa. The solid blue line represents the cumulative number of LLINs distributed since the Roll Back Malaria Program was launched. (*b*) The cumulative LLINs distribution in East and West/Central Africa from 2004 to 2020. We have used global distribution to derive the time series 2004–2015 in (*b*) (see Section S1 in electronic supplementary material). Data adopted from [18,19].

An estimated 552 million ITNs (or LLINs) were distributed by National Malaria Programs globally, with 83% of these delivered in sub-Saharan Africa (SSA) region from 2015 to 2017 [1]. Despite this massive scale-up in ITN distribution in SSA, malaria still persists in this region. One of the major reasons for the persistence of malaria in this region includes decay in ITN efficacy over time (e.g. due to adaptive mosquito behaviour and resistance, net attrition, and both natural and chemical decay) [5,6] and community-wide misuse of ITNs, e.g. for fishing, fencing and nursing of seedlings [7,8]. Individual preferences for ITN use evolve over time. Although the primary use of ITNs includes sleeping under them to avoid mosquito bites, they are used for other purposes such as agriculture and fishing as the ITN’s efficacy begins to wane [7,9,10]. Many studies have highlighted the gap between ITN ownership and ITN usage [11,12]. In particular, ITN usage is generally higher in high malaria transmission areas [11,13] during the wet season, but relatively lower during the dry season. In addition to seasonal variation, socio-economic factors (e.g. the comparative gain in daily productivity by using ITNs in agriculture, fishing or fencing), risk perception of malaria infection and severity, and the mosquitoes' density in the community are important determinants of ITN owners’ individual decisions to use ITNs properly [14]. Irrational use of ITNs is also well-documented. Several factors influence this—low mosquito activity [15], hot weather [14] and intention to achieve livelihood outcomes such as food security and increased household income through fishing [16–18]. Surveys have revealed that using ITNs for fishing reduces gross ITN coverage, impacting the effectiveness of anti-malarial campaigns [7,16]. Figure 1 shows a steady rise in the cumulative distribution of LLINs globally and the cumulative number of first observations of mosquito net fishing (MNF) usage from 1980 to 2015. This MNF survey study is a part of the Alliance for Malaria Prevention Net Mapping project distribution, which is available from 2004 [18]. Many mathematical models have studied the impact of ITN use and ITN efficacy on the spread and control of malaria [6,20–25]. Assuming a constant rate of effectiveness for the net’s lifespan, Chitnis *et al.* [23] demonstrated that ITNs are more effective than indoor residual spraying (IRS). Ngonghala *et al.* [25] introduced a differential equation-based model of ITN use, and concluded that ITN usage has a positive impact on reducing malaria transmission. Ngonghala *et al.* [26] investigated the interplay between ITNs, mosquito demography and mosquito resistance to insecticides used in ITNs. They showed that high ITN coverage and ITNs that retain their efficacy longer are better strategies to fight malaria in endemic regions. Apart from these, there are only handful of works that highlights human behaviour and ITN uses. For example, Honjo & Satake [27] developed an *N*-player game of ITN uses in community, and found Nash equilibrium depending on probability of malaria infection, poverty and other determinants. Broom *et al.* [28] also developed a game model of bed net use. However, none of these studies accounts for the evolution of the feedback process between ITN use and malaria prevalence interacting with behavioural, epidemiological and demographic parameters of the system. Understanding the combined impact of declining ITN efficacy and individual human decisions with respect to proper ITN usage is important, and a more complex dynamically coupled model of the evolution of ITN usage and malaria level may be needed to design and implement effective malaria control measures across communities in Africa and elsewhere.

In this study, we explore the impact of behavioural interactions combining evolutionary game theory and compartmental disease prevalence model. Game theory provides a useful tool for analysing behavioural interactions in disease control and prevention such as vaccination, social-distancing, treatment-seeking, self-medication and so on [29–35]. Here, we propose a mathematical model of individual adaptive behaviour in ITN usage that incorporates feedback from disease prevalence, declining efficacy of ITNs and mosquito population dynamics. Our analysis shows that highly efficacious ITNs may not be always sufficient for effective malaria control—an externality of human choices in ITN usage. This is an important and significant finding that cannot be obtained analysing a non-behavioural model. Furthermore, we show that sensitivity to disease prevalence and faster social learning can trigger cyclic oscillations that increase average ITN use rapidly. We compute social optimum ITN usage, which is a function of malaria transmission potential, ITN efficacy and mosquito demography. Under specific parameter regimes, our model also exhibits similar ITN-usage patterns observed in some regions of Africa. The study indicates that offering financial incentives in response to inappropriate ITN usage may help in preventing ITN misuse and contribute positively to malaria control efforts in Africa and elsewhere.

## 2. Modelling framework

### 2.1. Epidemiological model

**Figure 2.**
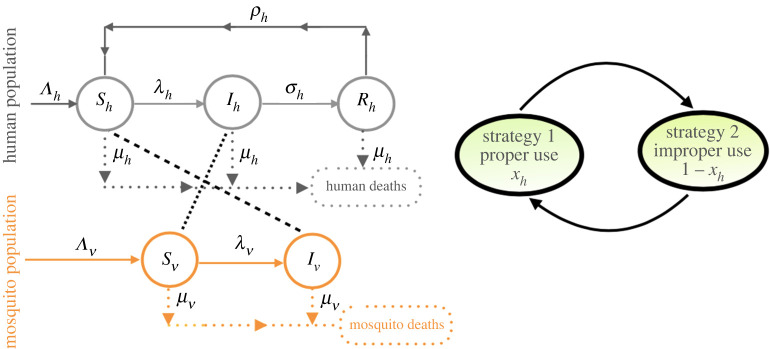
Schematic of the disease model (2.1) depicting transitions of humans and mosquitoes between different compartments (solid lines), transmission of malaria from infectious mosquitoes to susceptible humans (dashed line), transmission of malaria from infectious humans to mosquitoes (dashed dotted line). Moralities are denoted by dotted lines. Switching between strategies of ITN usages defines a feedback system—a part of behaviour-prevalence model (right panel of the figure).

The human population is divided into three compartments consisting of susceptible (*S*_*h*_), infectious (*I*_*h*_) and immune (*R*_*h*_) individuals. The mosquito population is divided into two classes, i.e. susceptible (*S*_*v*_) and infectious (*I*_*v*_) mosquitoes. With these divisions, the total human (*N*_*h*_) and mosquito (*N*_*v*_) populations are given by *N*_*h*_ = *S*_*h*_ + *I*_*h*_ + *R*_*h*_ and *N*_*v*_ = *S*_*v*_ + *I*_*v*_, respectively. In formulating the disease model, we assume that malaria cannot be transmitted vertically or horizontally. That is, all new human and mosquito births are susceptible and there is no direct human-to-human or mosquito-to-mosquito transmission. Schematics of the model are presented in figure 2, while brief descriptions of the model parameters and baseline and ranges of values of the model parameters are presented in table 1. The epidemiological model is described by the system of first-order ordinary differential equations

2.1 $$\begin{array}{ccc} & {\dot{S}}_{h}= & \Lambda_{h}-\lambda_{h}S_{h}+\rho_{h}R_{h}-\mu_{h}S_{h},\\ & {\dot{I}}_{h}= & \lambda_{h}S_{h}-A_{11}I_{h},\\ & {\dot{R}}_{h}= & \sigma_{h}I_{h}-A_{22}R_{h},\\ & {\dot{S}}_{v}= & \Lambda_{v}-\lambda_{v}S_{v}-\mu_{v}(t)S_{v}\\ \mathrm{and}\; & {\dot{I}}_{v}= & \lambda_{v}S_{v}-\mu_{v}(t)I_{v}, \end{array}\}$$

where *A*_11_ = *δ*_*h*_ + *σ*_*h*_ + *μ*_*h*_, *A*_22_ = *ρ*_*h*_ + *μ*_*h*_, and the forces of infection *λ*_*h*_ and *λ*_*v*_ as defined in [26] are

$$\lambda_{h}=\frac{\;p_{h}\beta I_{v}}{N_{h}}\;\text{and}\;\lambda_{v}=\frac{\;p_{v}\beta I_{h}}{N_{h}}.$$

The parameter, *p*_*h*_ (respectively, *p*_*v*_) is the probability that an infectious mosquito infects a susceptible human (respectively, the probability that an infectious human infects a susceptible mosquito) during a blood meal. The parameter *β* relates to the biting rate of mosquitoes defined below. Here, *x*_*h*_ is the fraction of population who use ITNs properly that prevent mosquito biting (see game-theoretic model below). Thus, proper usage of ITNs reduces the mosquito biting rate in the population, and the protection depends on the efficacy of ITNs (*b*_*β*_(*t*)).

**Table 1.** Descriptions, baseline values and references of parameters for (2.9)

parameter	description and dimension	baseline value	range	reference
$\Lambda_{h}$	recruitment rate of human by natural birth, respectively	10^3^/(55 × 365)	[2.7 × 10^−5^, 1.4 × 10^−4^]	[26] and refs therein
$\Lambda_{v}$	recruitment rate of mosquitoes by natural birth, respectively	0.145	[0.020, 0.27]	[26] and refs therein
*μ*_*h*_	human natural death rate (day^−1^)	1/(55 × 365)	[1/72, 1/35] × 1/365	
$\mu_{v_{0}}$	mosquito natural death rate (day^−1^)	1/14	[1/21, 1/14]	[26] and refs therein
$\mu_{v_{1}}$	pyrethroids-dependent death rate of mosquito	1/14	[1/21, 5/10]	[26] and refs therein
*δ*_*h*_	disease-induced human mortality rate (day^−1^)	32.9/(365 × 10^3^)	[0, 41/10^5^]	[26] and refs therein
*ρ*_*h*_	the rate humans loose malaria immunity (day^−1^)	1/(5 × 365)	[55/10^6^, 11/10^3^]	[26] and refs therein
*σ*_*h*_	rate at which infectious humans acquire immunity (day^−1^)	1/285	[14/10^4^, 17/10^3^]	[26] and refs therein
*p*_*v*_	the probability that a bite to an infectious human will infect a susceptible mosquito	0.48	[0.072, 0.64]	[26] and refs therein
*p*_*h*_	the probability that a bite of an infectious mosquito will infect a susceptible human	0.022	[0.01, 0.27]	[26] and refs therein
*β*_0_	mosquito biting rate per day	—	[1/10, 1]	[26] and refs therein
*b*_0_	implementation and efficacy parameter of personal protection by ITNs	—	[0, 1]	[26] and refs therein
*κ*	the imitation rate per day	—	[0.001, 0.2]	calibrated
*L*	the daily baseline productivity of an individual	1		calibrated
*r*	the related cost of using ITN properly	1	[8/10,18/10]	calibrated
*w*_1_	sensitivity parameter to infected human	1/100	—	calibrated
*w*_2_	sensitivity parameter to number of mosquitoes	1/6000	—	calibrated

#### 2.1.1. Decaying insecticide-treated net efficacy and periodic replacement

We follow the approach in [26] to model the function *b*_*β*_(*t*) for decaying ITN efficacy and ITN replacement by

2.2 $$\beta (b_{\beta }(t),x_{h})=\beta_{0}-\beta_{0}x_{h}b_{\beta }(t)$$

and

2.3 $$b_{\beta }(t)=\frac{2^{n}+1}{2^{n+1}}\left[\frac{2^{n}-1}{2^{n}+1}+\frac{1}{1+{\left((t\text{ mod }T)/(T/2)\right)}^{n}}\right]b_{0},$$

where *β*_0_ is an average mosquito biting rate that a human receives per day and 0 ≤ *b*_0_ ≤ 1 is the initial ITN efficacy. *n* determines shape of the efficacy curve, and *T* denotes the time interval replacing ITNs. It should be mentioned that *b*_0_ = 0 represents zero efficacious ITNs while *b*_0_ = 1 depicts full efficacy. Similarly, mosquito mortality rate $\mu_{v}(b_{\nu }(t),x_{h})$ is defined as [26]

2.4 $$\mu_{v}(b_{\mu_{v}}(t),x_{h})=\mu_{v_{0}}+\tilde{\mu_{v}}=\mu_{v_{0}}+x_{h}\mu_{v_{1}}(b_{\mu_{v}}(t))$$

and

2.5 $$b_{\mu_{v}}(t)=\frac{2^{n}+1}{2^{n}}\left[-\frac{1}{2^{n}+1}+\frac{1}{1+{\left((t\text{ mod }T)/(T/2)\right)}^{n}}\right]b_{0},$$

where $\mu_{v_{0}}$ represents natural mortality rate of mosquitoes and $\mu_{v_{1}}$ is the ITN-induced mosquito mortality rate. It is worth noting that $\lim_{t\to 0^{+}}\;b\mu_{1}(t)=b_{0}$, while $\lim_{t\to T^{-}}\;b\mu_{1}(t)=0$. This demonstrates that, when insecticides fail to kill mosquitoes, *μ*_*v*_ reduces to the natural mortality rate $\mu_{v_{0}}$. These functional forms are illustrated in figure S1 of the electronic supplementary material.

### 2.2. Game-theoretic model

We define this decision-making framework as a population game where the pay-off to each individual is determined by the individual’s strategy and the average behavioural strategy used by the population as a whole. Let *x*_*h*_, (0 ≤ *x*_*h*_ ≤ 1) be the proportion of population who adopt *strategy 1*, i.e. use ITN properly to protect themselves from mosquito bites. Then remaining 1 − *x*_*h*_ use ITNs for other purposes perceiving them to be more beneficial (*strategy 2*). As the individual choice of usage depends on the current disease prevalence, the players of the current generation not only play with each other but also play against players from previous generations with identical behaviours. In such strategic decision-making and social interaction, we assume that individuals emulate others’ activities. Specifically, they sample other members randomly at a constant rate, and if the pay-off of the sampled person is higher, then the sampled strategy is adopted with a probability that is proportional to the expected gain in pay-off [29,36]. It is assumed that individuals switch between the two strategies depending on the perceived benefits either from using ITNs properly or improperly.

In game theory, individuals act rationally in choosing the strategy that results in a higher pay-off. After an individual has access to an ITN, the individual selects the most preferred strategy that maximizes the associated expected utility. This adaptive behaviour is influenced by several social and economic factors. Let *L* ∈ [0, ∞) denote the baseline daily productivity of an individual. Then improper use of ITNs, e.g. for fishing or agriculture may increase baseline daily productivity. Let *r*_*L*_ > 1 be the proportional increment in the daily productivity. Then the perceived pay-off for improper ITN use is given by

2.6 $$f_{im}=L(r_{L}+1).$$

By contrast, proper use of ITNs reduces the risk of infection through mosquito bites and depends on the proportions of current disease prevalence (*I*_*h*_) and mosquito density (*N*_*v*_) in community. If *r*_*i*_ is the risk of infection, then the expected pay-off for proper use of ITNs is given by

2.7 $$f_{\;p}=r_{i}[1-b_{\beta }(t)](w_{1}I_{h}+w_{2}N_{v}),$$

where *w*_1_ and *w*_2_ are proportionality constants. It should be noted that the perceived pay-off is also a function of ITN efficacy since individuals are aware of the efficacy of ITNs from the onset. Thus pay-off gain for switching to the strategy of proper use of ITN is given by Δ*G* = *f*_*p*_ − *f*_*im*_ and accordingly, the evolution equation of *x*_*h*_ (when Δ*G* > 0) is given by

2.8 $$\begin{array}{rl} {\dot{x}}_{h} & =\varrho x_{h}(1-x_{h}).\varsigma \Delta G,\\ & =\kappa x_{h}(1-x_{h})\{-r+[1-b_{\beta }(t)](w_{1}I_{h}+w_{2}N_{v})\}, \end{array}$$

where $\kappa =\varrho \varsigma r_{i}$ is the scaled emulation or imitation rate, and *r* = *L*(*r*_*L*_ + 1)/*r*_*i*_ denotes the relative profit of improper usage of ITNs. This equation is similar to the replicator equation in population game [37]. It should be noted that fraction of individuals (1 − *x*_*h*_), who embark on the improper ITN use, satisfies the same equation (2.8).

### 2.3. Integrated epidemiological game-theoretic model

We now integrate the epidemiological model given by equations (2.1) and the game-theoretic model given by (2.8) into the coupled framework

2.9 $$\begin{array}{ccc} & {\dot{S}}_{h}= & \Lambda_{h}-\lambda_{h}S_{h}+\rho_{h}R_{h}-\mu_{h}S_{h},\\ & {\dot{I}}_{h}= & \lambda_{h}S_{h}-A_{11}I_{h},\\ & {\dot{R}}_{h}= & \sigma_{h}I_{h}-A_{22}R_{h},\\ & {\dot{S}}_{v}= & \Lambda_{v}-\lambda_{v}S_{v}-\mu_{v}(t)S_{v},\\ & {\dot{I}}_{v}= & \lambda_{v}S_{v}-\mu_{v}(t)I_{v}\\ \mathrm{and}\; & {\dot{x}}_{h}= & \kappa x_{h}(1-x_{h})\{-r+[1-b_{\beta }(t)](w_{1}I_{h}+w_{2}N_{v})\}. \end{array}\}$$

This model (2.9) is analysed and also numerically simulated further to investigate the impact of behavioural and epidemiological parameters on the dynamics of disease-prevalence model.

## 3. Results

### 3.1. Reproduction numbers and equilibrium

In this section, we explore the dynamics of the system (2.9) when ITN efficacy is constant, i.e. $b_{\beta }=b_{\mu_{v}}=b_{0}$. Thus, we set *β*(*t*) = *β*_0_ − *β*_0_*x*_*h*_*b*_0_ and $\mu_{v}(t)=\mu_{v_{0}}+\mu_{v_{1}}x_{h}b_{0}$. We note that in the absence of the malaria disease, the total human population $N_{h}(t)\to \Lambda_{h}/\mu_{h}$ as *t* → ∞ and the total mosquito population $N_{v}(t)\to \Lambda_{v}/\mu_{v}$ as *t* → ∞. It can be shown that the biologically feasible region $\Omega \subset {R^{6}}_{+}$, where

3.1 $$\Omega =\left\{(S_{h},I_{h},R_{h},S_{v},I_{v},x_{h})\in {R^{6}}_{+}\;:\;0\leq N_{h}(t)\leq \frac{\Lambda_{h}}{\mu_{h}},0\leq N_{\nu }(t)\frac{\Lambda_{v}}{\mu_{\nu }(b_{\mu_{\nu }})}\right\},$$

is a positively invariant, and hence, the model (2.9) is well-posed.

#### 3.1.1. Disease-free equilibrium

The model (2.9) has two *disease-free equilibria*—a full improper ITN-use equilibrium *E*_00_, which occurs when $x_{h}^{*}=0$, and a full proper ITN-use equilibrium *E*_01_, which occurs when $x_{h}^{*}=1$. We compute the basic reproduction number $R_{0}$ using the next-generation matrix approach [38]

3.2 $$R_{0}^{2}=\frac{{\beta_{0}}^{2}p_{v}p_{h}\mu_{h}\Lambda_{v}}{\mu_{v_{0}}^{2}\Lambda_{h}A_{11}}.$$

It can be shown that the disease-free equilibrium *E*_00_ is locally asymptotically stable when $R_{0}<1$ and unstable when $R_{0}>1$. It should be noted that the disease-free equilibrium *E*_01_ is unstable as there is always free riding when disease prevalence is low at the high ITN usages.

#### 3.1.2. Endemic equilibrium

The model has *endemic equilibrium* $E_{e}=(S_{h}^{*},I_{h}^{*},R_{h}^{*},S_{v}^{*},I_{v}^{*},x_{h}^{*})$, which gives the threshold quantity

3.3 $$R_{c}^{2}=\frac{\beta_{0}^{2}{\left(1-x_{h}^{*}b_{\beta }\right)}^{2}p_{v}p_{h}\mu_{h}\Lambda_{v}}{{\left(\mu_{v_{0}}+\mu_{v_{1}}x_{h}^{*}b_{\mu_{v}}\right)}^{2}\Lambda_{h}A_{11}},$$

as the control reproduction number. This is the expected number of secondary-infection cases introduced in a completely susceptible population in which a proportion of the population uses ITNs properly throughout the infectious period of the case. Depending on other parameters and $R_{c}$, the model has either single, or two, or even no endemic equilibrium. Detailed computation is given in the appendix.

### 3.2. Optimum insecticide-treated net use and malaria elimination

Since a strong nonlinear relationship exists between ITN efficacy and ITN use in a model where human behaviour is accounted for, it is important to investigate whether a *community-level optimum* ITN use exists for malaria elimination and whether it can be achieved under such individual adaptive social behaviour. Community-level optimum ITN usage is the limit or threshold, above which the *per capita* mosquito biting rate decreases and malaria prevalence decreases in the population and eventually eliminates from the population. Using expression of control reproduction number for the model (2.9) with constant ITN efficacy (*b*_0_) given by equation (3.3), we compute the optimum usage ${x_{h}}^{\mathrm{opt}}$, by setting ${x_{h}}^{*}={x_{h}}^{\mathrm{opt}}$ and solving for ${x_{h}}^{\mathrm{opt}}$. This gives

3.4 $${x_{h}}^{\mathrm{opt}}=\frac{1}{b_{0}}(\frac{K\beta_{0}-\mu_{\nu_{0}}}{K\beta_{0}+\mu_{\nu_{1}}}),\;\mathrm{where}\;K=\sqrt{\frac{\;p_{h}p_{v}\Lambda_{v}\mu_{h}}{\Lambda_{h}(\delta_{h}+\mu_{h}+\sigma_{h})}}.$$

The expression of $x_{h}^{\mathrm{opt}}$ in (3.4) defines an inverse relation with ITN efficacy. We plot the nonlinearity using a contour diagram in electronic supplementary material, figure S6 under three different ITN efficacies: (a) constant efficacy, (b) averaged efficacy, and (c) decaying efficacy with periodic replacement. As seen in the figure, the higher the efficacy, the better the chance of malaria elimination. However, the parametric regime of elimination is large under constant efficacy, but lowest when efficacy declines and ITNs are replaced periodically. We also plot the optimum threshold under different transmission potential (electronic supplementary material, figure S7). With higher transmission or biting rate the threshold increases monotonically depending on the value of ITN efficacy, implying that public health needs to increase much public awareness to increase bed net coverage especially in highly malaria-prevalent areas.

In the next few sections, we numerically simulate the model (2.9) to explore the impact of social learning and risk perception, decay in ITN efficacy, and financial incentives under different parameter regimes on malaria prevalence. Unless otherwise stated, the baseline parameter values in table 1 are used for the simulations. Some of the simulations here will involve the model (2.9) with decaying ITN efficacy (i.e. with $b_{\beta }$ and $b_{\mu_{v}}$ given by equations (2.3) and (2.5)), while others consider a constant ITN efficacy, i.e. $b_{\beta }=b_{\mu_{v}}=b_{0}$.

### 3.3. Impact of human behaviour and insecticide-treated net use on malaria dynamics

**Figure 3.**
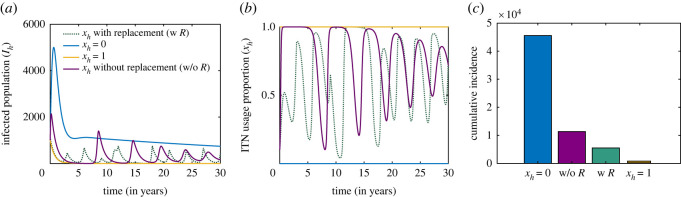
Malaria dynamics under mandatory and voluntary ITN use. Long-term dynamics of the (*a*) infectious human population and (*b*) proportion of proper ITN use under different ITN-usage scenarios. While there is high cumulative incidence with no ITN usage, the incidence is zero with complete proper ITN use. Continuous ITN usage without replacement exhibits regular oscillations, but ITN use with replacement every 3 years leads to an erratic pattern. (*c*) Cumulative infectious human population under the four scenarios *x*_*h*_ = 0, *x*_*h*_ = 1, 0 < *x*_*h*_ < 1 with and without ITN replacement. The initial conditions used for the simulations are $\left(S_{h}^{0},I_{h}^{0},R_{h}^{0},S_{v}^{0},I_{v}^{0},x_{h}^{0})=(10\;000,1000,0,20\;000,4000,0.01\right)$, *b*_0_ = 0.75, *β*_0_ = 0.5, *κ* = 0.01 and the other parameter values are presented in table 1.

Figure 3 depicts disease dynamics under different ITN-usage scenarios. The model exhibits a stable dynamics when there is completely no ITN use, i.e. when *x*_*h*_ = 0 (blue curve in figure 3*a*) and disease prevalence is almost zero when everybody compulsorily uses ITNs properly, i.e. when *x*_*h*_ = 1 (golden curve in figure 3*a*). However, the qualitative and quantitative pattern of prevalence changes when ITN usages are considered as individual interest (given by model equations (2.9)). In the mixed strategy scenario with no replacement case (i.e. constant efficacy), the system settles down to a relatively stable prevalence state through decaying oscillations due to individual social learning and imitation in adapting strategies (purple curve in figure 3*a*). In this case, the proportion of ITN users also varies between 0 and 1 with decaying oscillation (purple curve in figure 3*b*). By contrast, while we have periodic ITN replacement, the disease prevalence is irregular in the beginning, but eventually settles on a bounded periodic pattern that synchronizes with ITN replacement (dotted green curve in figure 3*a*). Individuals switch to proper ITN use immediately after replacement due to the high efficacy but eventually refrain from proper use when disease prevalence is low. It should be noted that high proper ITN use lowers the disease prevalence that eventually reduces the disease risk. This motivates individuals to free ride, causing improper usage of ITNs. These, however, lead to complex patterns in malaria prevalence and different cumulative incidence of malaria (figure 3*c*). Thus, behavioural interaction plays an important role in predicting malaria prevalence dynamics, which is important from a public health point of view.

### 3.4. Impact of individual social learning and risk perception on insecticide-treated net use and malaria prevalence pattern

**Figure 4.**
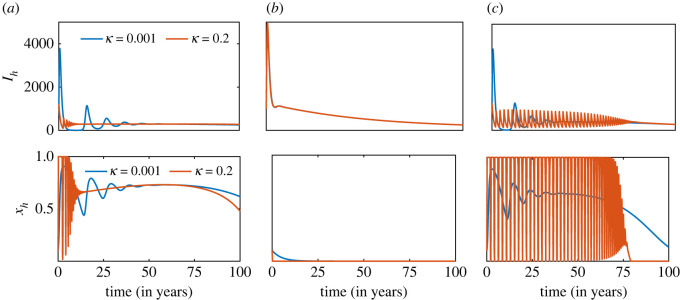
Impact of social learning and perception pertaining to ITN use. Malaria incidence (upper panel) and the proportion of proper ITN use (lower panel) when the perceived probability of infection depends on (*a*) only malaria prevalence, (*b*) only mosquito density and (*c*) both malaria prevalence and mosquito density. Each scenario is investigated for a low (blue curve) and high (red curve) imitation rate value and without periodic ITN replacement. The initial conditions, *b*_0_, *β*_0_, are the same as those for figure 3, and the other parameter values used for the simulations are presented in table 1.

Individual social learning and perception in estimating the cost of ITN usage have a significant impact on the prevalence pattern of malaria. Since there is no empirical data describing how individuals perceive the risk of malaria infection and severity, and calculate the pay-off for proper ITN use, we consider three different scenarios: (i) malaria prevalence (*w*_1_ ≠ 0, *w*_2_ = 0), (ii) mosquito density (*w*_1_ = 0, *w*_2_ ≠ 0), and (iii) both malaria prevalence and mosquito density (*w*_1_ ≠ 0, *w*_2_ ≠ 0) to construct the pay-off function for the game dynamics, and illustrate the impact of proper ITN use on disease transmission. Each scenario is considered for two imitation rates (*κ* = 0.001 and *κ* = 0.2) and ITN efficacy is constant, i.e. $b_{\beta }=b_{\mu_{v}}=b_{0}$. The results obtained and presented in figure 4 shows that small transient oscillations followed by stable dynamics in case (i) (figure 4*a*), while there is a huge infection wave followed by a stable prevalence when the perception of infection risk depends only on mosquito density (case (ii) (figure 4*b*). When risk perception depends on both malaria prevalence and mosquito density (case (iii)), the model exhibits low amplitude but very high-frequency oscillations and eventually settles on a very low stable prevalence level in the long run (figure 4*c*). There is frequent switching between strategies, and entire populations switch from improper ITN use to proper ITN use as disease prevalence starts rising in the population. This contrasting pattern in the dynamics occurs because the total mosquito density in the community remains stable for a long time. This impedes frequent changes in individual risk perceptions, thereby inhibiting switching between strategies. By contrast, sensitivity to only malaria prevalence influences individual risk perceptions and hence results in quick switching to proper ITN use. But once disease prevalence becomes low, individuals abandon proper ITN use and switch to improper use. The observed oscillatory dynamics are due to the imitation rate from the game-theoretic component of the model, which regulates strategy switching. In particular, higher social learning motivates individuals to imitate others and switch between strategies quickly if it provides better pay-off, while low imitation and social learning retards strategy switching. The cumulative incidence is higher when the perceived risk of infection depends only on mosquito density.

Additional simulations were carried out for periodic ITN replacement, i.e. the case in which the model $b_{\beta }$ and $b_{\mu_{v}}$ are given by equations (2.3) and (2.5). The results obtained and presented in figure S4 in the electronic supplementary material show similar dynamics to those for the case with constant ITN efficacy, except for the scenario in which risk perception depends on malaria prevalence. Specifically, disease prevalence is almost zero with full proper ITN use when the imitation rate, *κ* = 0.001 (electronic supplementary material, figure S4(a)). Since ITN efficacy declines and ITNs are replaced periodically, switching between strategies occurs more interactively over time. However, the qualitative pattern of ITN usage at higher imitation rates shows erratic oscillations, which induce periodic oscillations in prevalence later (electronic supplementary material, figure S4(c)).

### 3.5. Impact of insecticide-treated net efficacy on malaria prevalence

**Figure 5.**
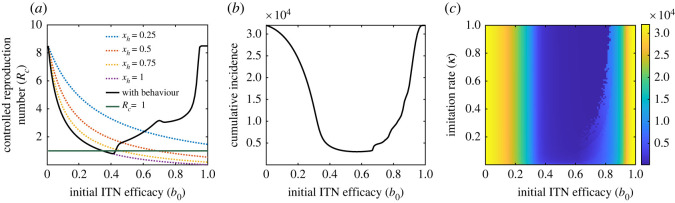
Impact of ITN efficacy on malaria burden. (*a*) Plot of the control reproduction number (*R*_*c*_) as a function of ITN efficacy (*b*_0_) for different proportions of mandatory and voluntary usages. Dotted curves denote *R*_*c*_ values for different proportions of mandatory use, while the solid black curve denotes *R*_*c*_ values for voluntary ITN use. (*b*) Cumulative incidence for different values of ITN efficacy under voluntary use of ITNs. (*c*) Heatmap of the cumulative malaria incidence as a function of the imitation rate (*κ*) and ITN efficacy. The region of minimum cumulative incidence for different values of *b*_0_ increases as the imitation rate increases. Here, $\mu_{v_{0}}=0.0156$, $\mu_{v_{1}}=0.21$, *β*_0_ = 0.3 and the other parameter values are presented in table 1.

Bed nets always serve as a physical barrier between humans and mosquitoes, and hence reduce the chance of mosquito biting. Proper use of bed nets not only provides personal protection from malaria infection but also extends to the community-level indemnity against the malaria infection. ITNs either disorient resting mosquitoes through their repellent effect or kills them. So, higher ITN coverage decreases the *per capita* biting rate by reducing the mosquito population in the community. However, higher ITN efficacy may not always lead to a reduction in malaria transmission, as individuals’ choices of using bed nets evolve depending on current disease prevalence and ITN efficacy. To assess the impact of ITN efficacy on overall malaria prevalence, we compute the *control reproduction number* *R*_*c*_ (equation (3.3)) against different values of ITN efficacy, considering different scenarios such as mandatory and voluntary use of bed nets. We first estimate *R*_*c*_ under different proportional mandatory use of ITNs. As seen in figure 5, there is a monotonic decreasing relationship between *R*_*c*_ and *b*_0_. In particular, if the proportion of susceptible humans who choose to use ITNs properly (*x*_*h*_) is as low as 25% (i.e. *x*_*h*_ = 0.25), *R*_*c*_ will never be below one (blue dotted curve in figure 5*a*). If half of the population use ITNs properly (i.e. if *x*_*h*_ = 0.5) then efficacy of 0.71% is required to reduce the control reproduction number below one (red dotted curve in figure 5*a*). Furthermore, if everyone decides to use ITNs properly (i.e. if *x*_*h*_ = 1.0) then the efficacy of ITNs as low as 34% is required to reduce the control reproduction number below one (purple dotted curve in figure 5*a*). By contrast, when ITN usages are taken as rational choices of humans, *R*_*c*_ falls below one only when ITN efficacy is within the interval (0.32, 0.48) (black curve in figure 5*a*). Hence, under this parameter regime, the disease can be contained for efficacy lying between 0.32 < *b*_0_ < 0.48. This implies that lower or higher efficacy of ITNs is not always useful in controlling the infection in the community. Out of this range, the control reproduction number is greater than one, rendering disease control difficult. Thus, although higher proportions of proper ITN usage in a community can lower *R*_*c*_ below one even at small values of *b*_0_, this might not be possible with very low or very high ITN efficacy when ITN use is considered a rational choice of individuals.

The model (2.9) is further simulated to assess the impact of ITN efficacy on the cumulative incidence of malaria for 20 years. The results (presented in figure 5*b*) show that the cumulative incidence of malaria is very low for 0.32 < *b*_0_ < 0.48. This concave pattern of cumulative incidence is also observed in cases with periodic ITN replacements (electronic supplementary material, figure S5). This pattern is due to the fact that at lower ITN efficacy, individuals ignore using ITNs, while at high ITN efficacy, disease transmission drops quickly and it remains no primary incentive for using ITNs properly. So, individuals either abandon ITN use or switch to improper use. As a consequence, there is an increase in cumulative prevalence. We further investigate this concave pattern of cumulative incidence under different values of imitation rates (where a higher imitation rate signifies faster switching) and different ITN-efficacy values. The results presented in figure 5*c* show that the region of minimum cumulative incidence shrinks as the imitation rate decreases. Faster responses bring the incidence to a minimum by lowering the transmission potential. Similar observations are found in electronic supplementary material, figure S5(b).

This analysis reveals that very low and high ITN efficacy is not practical in reality, as human behaviour is an inevitable component in evaluating the impact of ITN usage in controlling malaria, especially in regions like SSA. It also indicates that risk communication and creating awareness about the benefits of ITNs by public health authorities to their communities should be an integral part of any malaria elimination strategy.

### 3.6. Social optimum insecticide-treated net use

It has frequently been observed that such rational, selfish behaviour by individuals within a population leads to suboptimal health outcomes [29]. We address it here in the ITN-usage game for malaria control. We compare the total burden of disease as well as daily productivity loss due to proper ITN use under the Nash equilibrium with the total burden under a defined socially optimal strategy that benefits the population as a whole. In this context, the social optimum is defined as ITN coverage that minimizes the total burden in the population from either loss of daily productivity or infection.

As both the actual costs of infection and actual loss in productivity are lower than the perceived cost, comparing the socially optimal coverage with Nash equilibrium is trivial under actual cost. By contrast, we demonstrate the scenario where both the social optimum and the Nash equilibrium are based on the individual-level perceived cost. Thus, we define the total burden as follows:

3.5 $$J(x_{h})=\int_{0}^{T}[r_{L}S_{h}x_{h}+r_{i}I_{h}]\;dt,$$

where *r*_*L*_ is *per capita* perceived loss due to proper use of ITN and *r*_*i*_ is perceived cost infection in a predefined time period.

**Figure 6.**
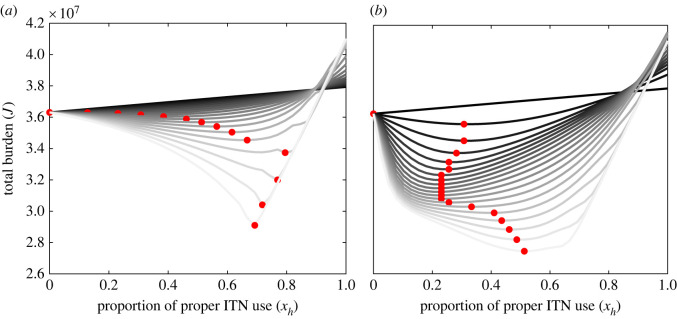
Social optimum ITN use and total burden. Total burden *J* as function of ITN uses *x*_*h*_ and (*a*) constant ITN efficacy *b*_0_, (*b*) periodic ITN efficacy with amplitude *b*_0_. Curves with shades of grey colour represent from *b*_0_ = 0 (dark black) to *b*_0_ = 1 (light grey). Red dots indicate the minimum burden at the social optimum ITN usages for respective ITN efficacy. Here *β*_0_ = 0.25, *p*_*h*_ = 0.22 and other parameter values are the same as given in table 1.

Similar outcomes are observed for both the cases—constant ITN efficacy, and declining ITN efficacy with periodic replacement of ITNs. Figure 6*a* plots the total burden under different values of constant ITN efficacy. As observed, with higher ITN efficacy the social burden either from infection or loss due to proper use is low, but it increases with low efficacy. However, in every aspect, the social optimum exists. The social optimum ITN usage increases with increasing the efficacy up to a certain threshold. High ITN efficacy minimizes the infection in the community even under low ITN coverage. By contrast, lower ITN efficacy increases the chance of infection in the community even after a high proportion of proper usage. This way, it increases the total burden. A similar observation is made in the case of declining efficacy with periodic replacement of ITNs every 3 years (figure 6*b*). In this case, the transmission potentiality changes every 3 years—low transmission at higher initial efficacy, and high transmission at lowest efficacy. However, the average efficacy determines the optimal ITN coverage in the population for minimum burden.

### 3.7. Impact of public health interventions and financial incentives

**Figure 7.**
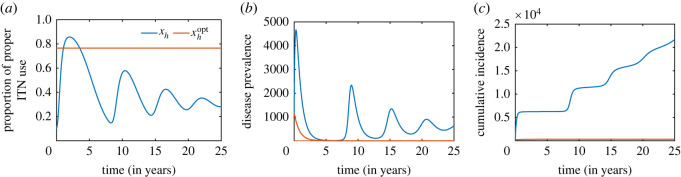
Comparison of dynamics between community-level optimum ITN use, and the use under individual interest (constant ITN efficacy (*b*_0_)). Plots of (*a*) proper ITN use, (*b*) disease prevalence (*I*_*h*_) and (*c*) cumulative infections. Here, *b*_0_ = 0.85, $\mu_{v_{1}}=0.5$, *p*_*h*_ = 0.22, *κ* = 0.002, *β*_0_ = 0.25 and the other parameters are as given in table 1. The initial conditions used for the simulation are $\left(S_{h}^{0},I_{h}^{0},R_{h}^{0},S_{v}^{0},I_{v}^{0},x_{h}^{0})=(10\;000,1000,0,20\;000,4000,0.01\right)$. A plot of the dynamics for a longer time period is given in electronic supplementary material, figure S8.

While community-level optimum ITN usages for malaria elimination depend on several disease-related parameters, we observe that the proportion of proper ITN use under individual interest (strategy 1) is below this community-level optimum under the baseline values of the parameters in table 1 (figure 7*a*). Consequently, the cumulative number of infections increase steadily under individual choice of proper ITN use (figure 7*c*).

**Figure 8.**
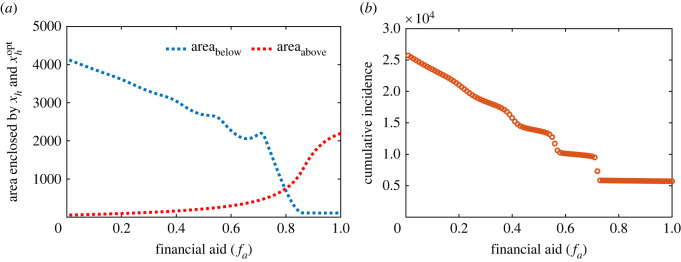
Impact of financial aid on ITN use and cumulative infection. (*a*) Area enclosed by *x*_*h*_ and $x_{h}^{\mathrm{opt}}$ when $x_{h}^{\mathrm{opt}}>x_{h}$ (dotted blue curve) and when $x_{h}>x_{h}^{\mathrm{opt}}$ (dotted red curve) as a function of financial aid (*f*_*a*_) over a time span of 30 years (see electronic supplementary material, figure S7 for an illustration). (*b*) Cumulative malaria incidence as a function of financial aid. Here, *b*_0_ = 0.85, $\mu_{v_{1}}=0.5$, *p*_*h*_ = 0.22, *κ* = 0.002, *β*_0_ = 0.25 and the other parameters are as given in table 1. The initial conditions used for the simulation are $\left(S_{h}^{0},I_{h}^{0},R_{h}^{0},S_{v}^{0},I_{v}^{0},x_{h}^{0})=(10\;000,1000,0,20\;000,4000,0.01\right)$.

This, however, demonstrates that there are significant disparities between population optimum and the interest of individuals to use ITNs properly. In this section, we investigate whether population optimum can be achieved under such adaptive social behaviour. Several studies have identified poverty, a decline in alternative resources, ease of access to ITNs, additional income etc. as the major driving factors for individuals to use their ITNs in fishing, agriculture and other activities instead of protection against mosquito bites [18,39]. Here, we assess the impact of financial incentives from local public health authorities or government as a measure to curb using ITNs for other economic-related activities on ITN use and malaria control by modifying the utility function (2.7) as follows:

3.6 $$\Delta G_{\;f}=-r+(1-b_{\beta }(t))(w_{1}I_{h}+w_{2}N_{v})+f_{a},$$

where 0 < *f*_*a*_ < 1 is financial aid. The modified model (2.9) with Δ*G*_*f*_ in the equation for *x*_*h*_ given by (3.6) is simulated for different values of *f*_*a*_ and the areas enclosed by the solution curve of individual interest above and below the social optimum value are computed. Electronic supplementary material, figure S9 gives a better representation of the enclosed areas. It shows that the area_above_ increases and area_below_ decreases with an increase in *f*_*a*_, implying that the fraction of proper ITN usages increases as the financial aid increases (figure 8*a*). Consequently, the cumulative number of infectious humans decreases (figure 8*b*). Furthermore, for higher values of *f*_*a*_ (approx. 0.8), individual interest increases and crosses the population optimum, triggering increases in the inter-epidemic period in malaria prevalence. Thus, financial incentives from local authorities or government can motivate more individuals to embark on proper ITN use and/or facilitate the attainment of the optimum coverage even under such adaptive behaviour of individuals. See electronic supplementary material for sensitivity analysis of this result (figure S10). This result provides some useful insights for the design of malaria control strategies in resource-challenged communities.

## 4. Empirical data and model implication

Our theoretical framework explains externalities to the community as a consequence of improper use of ITNs, and discusses the implications of feedback in the dynamics due to public health and governmental support. The feedback loop formed by disease prevalence and rational decisions by individuals on proper ITN use explains the failure of malaria elimination efforts and the persistence of malaria in some parts of the world, e.g. in SSA. Empirical testing of such feedback loops or parameter estimation for such reinforcing dynamics requires high-resolution longitudinal data on disease transmission, ITN distribution, proper and improper ITN use, and economic growth at both the population and individual levels. To our knowledge, such data are rarely available in public repositories. Hence, testing our theory is challenging. However, we use our model to test a more approachable goal by predicting the pattern of improper use of ITNs observed in the aggregated data of mosquito net fishing (MNF) in both East and West/Central Africa (figure 1*a*).

**Figure 9.**
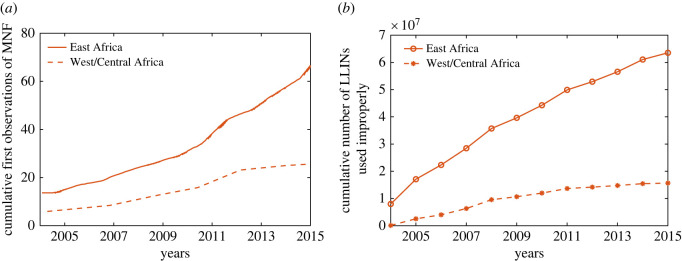
Empirical observation and model projection. (*a*) Cumulative number of first observations of mosquito net fishing (MNF) from [18]. (*b*) Cumulative number of individuals using ITNs improperly obtained from the model (2.9). Observe that the pattern of improper ITN use from our theory model is similar to that from the East African and West/Central African data. The calibrated parameters of the model (2.9) using the East African data are *β*_0_ = 0.2, *κ* = 0.0006, while the calibrated parameters of the model (2.9) using the West and Central African data are *β*_0_ = 0.3, *κ* = 0.0009. The other parameters are discussed in the electronic supplementary material.

To test our theoretical model, we extracted the global distribution of LLINs from figure 1*a*,*b* [18] and obtained the year-wise distribution of ITNs in East and West/Central Africa figure 9*a*. This year-wise distribution data is then fed into our model in order to determine the improper ITN use from the output variables of the model (see Section S1 of the electronic supplementary material for details of the implementation). Using different values for the disease transmission rate and social learning (i.e. imitation rate) in the population, we obtained the cumulative improper ITN use from simulations of the model (2.9). The obtained results depicted in figure 9*b* show that the pattern of improper ITN use increases over time, as the distribution of ITNs increases yearly, which is consistent with the qualitative pattern in the observed data. We have also performed univariate and multivariate analysis of the results (see the electronic supplementary material for more details (figure S11)).

## 5. Discussion and conclusion

In almost every high malaria-endemic country in Africa and Asia, ITNs have been found to be the most applicable and cost-effective malaria preventive intervention [40]. Regular use of ITNs in Africa has resulted in a 20% reduction in child mortality, and a substantial reduction in the impact of malaria morbidity on children and pregnant women [41]. When ITNs are widely used in a community, they protect not only those who sleep under them, but also those in the same dwelling and nearby communities (called *community effect*). Promoting rapid and sustained scale-up of ITN use is thus a critical approach to reducing malaria morbidity and mortality. However, there is significant uncertainty in ITN use, characterized by spatial and temporal variability, which poses potential challenges for malaria control programmes. [42]. Many studies have highlighted the contrariety between ownership and effective usage of ITNs in various communities [12,43]. For example, cross-sectional surveys on ITN ownership (possession), compliance (proper usage among those who own ITNs) and malaria infections among occupants were carried out in selected highland areas in Western Kenya in 2009 [11]. The results of the survey revealed that although ITN ownership was more than 71%, less than half of the population used the ITNs. The survey also revealed that the compliance rate was also varied seasonally—higher during the rainy season compared with the dry season. The conclusions are similar to those of other studies conducted in Niger, Mali, Ghana, Ethiopia, Zambia and other SSA countries [12]. Thus, the potential use of ITNs to protect humans from mosquito bites is a crucial issue in the malaria elimination efforts in African countries. Therefore, understanding human behaviour in ITN use, and intra-household net-use patterns, can assist malaria control programmes in effectively directing their efforts to achieve increased public health impact.

In this manuscript, we develop a game-theoretic framework to understand human decisions in ITN use. There are several coevolving determinants behind an individual’s rational decision in ITN usages such as ITN efficacy, malaria prevalence, mosquito density, temperature and seasonal factors. On the other hand, the use of ITNs in agriculture and fishing or for fencing increases daily economic output for some families [9,18]. As a result, ITN use changes in response to the actual pay-offs to individuals, which are determined by disease prevalence, mosquito density in the same dwelling or community, current efficacy of ITNs and benefit from using ITNs for other purposes. This feedback loop between individual choice and disease transmission is an adaptive social learning process in which the community modifies ITN use in response to disease prevalence. This study shows how prompt individual responses to underlying disease prevalence or mosquito density affect coevolutionary dynamics. Slow social learning and sensitivity to only mosquito density lead to stable but high prevalence, whereas with faster imitation and sensitivity to both disease prevalence and mosquito density, individuals frequently switch to other strategies that interrupt disease transmission and slow down the growth of infections in the community. In the latter case, individuals adopt proper ITN use so quickly that the community settles on a social norm that reaches the population optimum ITN use.

The optimum threshold of ITN usage for malarial elimination is an important issue for public health authorities. Our model shows that the ITN coverage from individual interest is lower than the optimum threshold in a population, but still can be achieved under this adaptive social behaviour. In fact, poverty among poor economic policies constitutes a predominant challenge in achieving the social optimum ITN usage. Many studies have highlighted malaria with poverty, which is apparent since it is highly prevalent mostly in regions with poor socio-economic conditions that prevent populations from using ITNs optimally [44,45]. A continuous effort is required to ensure the development and implementation of policies that promote rapid and sustained economic growth, as well as programmes to improve the livelihoods of individuals in impoverished malaria communities [46]. This study shows that financial incentives to support household resources and other activities can change people’s perceptions of inappropriate ITN use and lead to an increase in proper ITN use.

Our theoretical results are in line with findings from several studies such as WHO’s *Roll Back Malaria* initiative that highlights appropriate balance between subsidized approaches and sustainable market development, targeting vulnerable groups and communities for scaling up ITN use in populations [47]. Other game-theoretic studies of ITN uses also draw similar conclusions [27,28]. Effective malaria elimination strategies can be achieved by engaging stakeholders from government and non-governmental institutions, private sector partners, public health officials, environmentalists, healthcare practitioners and research institutions [18]. In addition, ITN awareness promotion at both individual and population level, as well as to implement subsidized control and economic schemes for vulnerable groups and communities are crucial.

The model has limitations due to simplified assumptions. Seasonality in mosquito density is an important aspect of such epidemic dynamics, and it has potential to influence individual ITN use [14]. An age-structured model may be more appropriate for policy-specific questions, because the risk of infection differs between children under the age of five and young and old people. [48]. Similarly, a detailed optimization model is preferable for developing strategic policies that integrate ITN distribution with a subsidy plan in order to increase ITN coverage in vulnerable communities. These behavioural models must also be tested and validated using empirical data, which is scarce in the literature. Despite the availability of malaria case notifications, empirical data on proper ITN use is not readily available in public repositories. Representative population-wide surveys detailing both quantitative and qualitative aspects of perceived gain in proper and domestic ITN use are required to advance understanding of human behaviour in ITN use.

## Data Availability

Data have been presented in the electronic supplementary material [49].

## Appendix A

**A.1. Reproduction numbers and disease-free equilibrium**

The model (2.9) has two disease-free equilibrium points—a full improper ITN-use equilibrium *E*_00_ which occurs when $x_{h}^{*}=0$, and a full proper ITN-use equilibrium *E*_01_, which occurs when $x_{h}^{*}=1$. These disease-free equilibrium points are given by

$$E_{00}=(S_{h}^{*},0,0,S_{v}^{*},0,0)=\left(\frac{\Lambda_{h}}{\mu_{h}},0,0,\frac{\Lambda_{v}}{\mu_{v_{0}}},0,0\right)$$

and

$$E_{01}=(S_{h}^{*},0,0,S_{v}^{*},0,1)=\left(\frac{\Lambda_{h}}{\mu_{h}},0,0,\frac{\Lambda_{v}}{\mu_{v_{0}}+\mu_{v_{1}}b},0,1\right).$$

**A.2. Endemic equilibrium**

Endemic equilibrium points of the model $E_{e}=(S_{h}^{*},I_{h}^{*},R_{h}^{*},S_{v}^{*},I_{v}^{*},x_{h}^{*})$ are given by

$$\begin{array}{rl} & S_{h}^{*}=\frac{\Lambda_{h}A_{11}A_{22}}{A_{h}^{*}},I_{h}^{*}=\frac{{\lambda_{h}}^{*}\Lambda_{h}A_{22}}{A_{h}^{*}},\\ & R_{h}^{*}=\frac{{\lambda_{h}}^{*}\Lambda_{h}\sigma_{h}}{A_{h}^{*}},S_{v}^{*}=\frac{\Lambda_{v}}{\lambda_{v}^{*}+\mu_{v}},\;I_{v}^{*}=\frac{\lambda_{v}^{*}\Lambda_{v}}{\mu_{v}(\lambda_{v}^{*}+\mu_{v})}\\ & x_{h}^{*}=\frac{1}{\mu_{v_{1}}b_{0}}[\frac{(1-b_{0})w_{2}\Lambda_{v}}{r-(1-b_{0})w_{1}I_{h}^{*}}-\mu_{v_{0}}],A_{h}^{*}=A_{11}A_{22}(\lambda_{h}^{*}+\mu_{h})-\lambda_{h}^{*}\sigma_{h}\rho_{h}>0, \end{array}$$

where

$$\lambda_{h}^{*}=\frac{\;p_{h}\beta \Lambda_{v}}{\mu_{v}}\frac{A_{11}A_{22}(\lambda_{h}^{*}+\mu_{h})-\lambda_{h}^{*}\sigma_{h}\rho_{h}}{\Lambda_{h}[A_{11}A_{22}+\lambda_{h}^{*}(A_{22}+\sigma_{h})]}\frac{\lambda_{v}^{*}}{\lambda_{v}^{*}+\mu_{v}}\;\mathrm{and}\;\lambda_{v}^{*}=\frac{\;p_{v}\beta \lambda_{h}^{*}A_{22}}{\left[A_{11}A_{22}+\lambda_{h}^{*}(A_{22}+\sigma_{h})\right]}.$$

Substituting the value of $\lambda_{v}^{*}$ from the first equation of (A 1) in the second equation and expanding in $\lambda_{h}^{*}$ yields the following polynomial equation:

$$(A_{2}{\lambda_{h}^{*}}^{2}+A_{1}\lambda_{h}^{*}+A_{0})=0,$$

where *A*_2_ = (*A*_22_ + *σ*_*h*_)[*p*_*v*_*βA*_22_ + (*A*_22_ + *σ*_*h*_)*μ*_*v*_], $A_{1}=(A_{11}A_{22})p_{v}\beta A_{22}+2(A_{11}A_{22})(A_{22}+\sigma_{h})\mu_{v}+(\beta^{2}p_{h}p_{v}\Lambda_{v}A_{22}/\mu_{v}\Lambda_{h})(\sigma_{h}\rho_{h}-A_{11}A_{22})$, $A_{0}=\mu_{v}{\left(A_{11}A_{22}\right)}^{2}(1-R_{c}^{2})$.

Solving equation (A 2) using the quadratic formula gives $\lambda_{h}^{*}=-A_{1}\pm \sqrt{A_{1}^{2}-4A_{0}A_{2}}/2A_{2}$. Depending on the sign of *A*_1_ and the discriminant $A_{1}^{2}-4A_{0}A_{2}$, $\lambda_{h}^{*}$ can have zero, one or two values. This leads to the following results:

The malaria-human behaviour model (2.9) with $b_{\beta }=b_{\mu_{v}}=b_{0}$, where *b*_0_ is constant has

(a) a single endemic equilibrium solution if any of the following three conditions are satisfied:
  (i) *A*_0_ < 0 or $R_{c}>1$
  (ii) *A*_1_ < 0 and *A*_0_ = 0, or
  (iii) *A*_1_ < 0 and $A_{1}^{2}-4A_{0}A_{2}=0$;
(b) two endemic equilibrium solutions if *A*_0_ > 0, *A*_1_ < 0 and $A_{1}^{2}-4A_{0}A_{2}>0$;
(c) no endemic equilibrium solution otherwise.
